# Road Traffic Noise and Incident Myocardial Infarction: A Prospective Cohort Study

**DOI:** 10.1371/journal.pone.0039283

**Published:** 2012-06-20

**Authors:** Mette Sørensen, Zorana J. Andersen, Rikke B. Nordsborg, Steen S. Jensen, Kenneth G. Lillelund, Rob Beelen, Erik B. Schmidt, Anne Tjønneland, Kim Overvad, Ole Raaschou-Nielsen

**Affiliations:** 1 Institute of Cancer Epidemiology, Danish Cancer Society, Copenhagen, Denmark; 2 Department of Public Health, University of Copenhagen, Copenhagen, Denmark; 3 Department of Environmental Science, Aarhus University, Roskilde, Denmark; 4 Rambøll Danmark A/S, Aarhus, Denmark; 5 Institute for Risk Assessment Sciences, Utrecht University, Utrecht, The Netherlands; 6 Department of Cardiology, Centre for Cardiovascular Research, Aalborg Hospital, Aarhus University Hospital, Aalborg, Denmark; 7 Department of Epidemiology, School of Public Health, Aarhus University, Aarhus, Denmark; Medical University Innsbruck, Austria

## Abstract

**Background:**

Both road traffic noise and ambient air pollution have been associated with risk for ischemic heart disease, but only few inconsistent studies include both exposures.

**Methods:**

In a population-based cohort of 57 053 people aged 50 to 64 years at enrolment in 1993–1997, we identified 1600 cases of first-ever MI between enrolment and 2006. The mean follow-up time was 9.8 years. Exposure to road traffic noise and air pollution from 1988 to 2006 was estimated for all cohort members from residential address history. Associations between exposure to road traffic noise and incident MI were analysed in a Cox regression model with adjustment for air pollution (NO_x_) and other potential confounders: age, sex, education, lifestyle confounders, railway and airport noise.

**Results:**

We found that residential exposure to road traffic noise (L_den_) was significantly associated with MI, with an incidence rate ratio IRR of 1.12 per 10 dB for both of the two exposure windows: yearly exposure at the time of diagnosis (95% confidence interval (CI): 1.02–1.22) and 5-years time-weighted mean (95% CI: 1.02–1.23) preceding the diagnosis. Visualizing of the results using restricted cubic splines showed a linear dose-response relationship.

**Conclusions:**

Exposure to long-term residential road traffic noise was associated with a higher risk for MI, in a dose-dependent manner.

## Introduction

Increasing noise from traffic occurs in parallel with urbanisation. Noise acts as a stressor provoking a typical stress response, with hyperactivity of the sympathetic autonomic nervous system followed by elevated blood pressure, heart rate and vasoconstriction [1]. Furthermore, noise affects the hypothalamus-pituitary-adrenal axis, leading to increased levels of cortisol [2]. In addition, exposure to noise during the night at normal urban levels has been associated with sleep disturbances [3], which might affect metabolic and endocrine function [4], [5] and impair the immune system [6], [7].

In 2008 a meta-analysis identified seven studies which had investigated the association between road traffic noise and myocardial infarction (MI) using objective noise exposure with multiple categories and control for confounding as inclusion criteria [8]. The study found no effect of noise in cross-sectional studies, whereas in case-control and cohort studies there seemed to be a higher risk for MI at exposures to noise levels above 60 dB, though statistically insignificant for all exposure groups. None of the studies in the meta-analysis included adjustment by air pollution, which might be important as road traffic noise and ambient air pollution correlate [9], [10] and both exposures have been linked to MI [8], [11]. Recently, two studies have investigated the joint effect of road traffic noise and air pollution on ischemic heart disease with mixed results [9], [12]. While Selander et al. found that road traffic noise seemed to increase the risk for incident MI after adjustment for air pollution (NO_2_), Beelen et al. found that after adjustment for black smoke and traffic intensity there was no association between road traffic noise and risk for mortality from ischemic heart disease.

The aim of the present study was to investigate the association between residential exposure to road traffic noise and risk for incident MI in a large prospective cohort after adjustment for exposure to air pollution and other important risk factors for MI.

## Methods

### Ethics Statement

The study was approved by the regional ethic committee for Copenhagen and Frederiksberg. Participation in the Diet, Cancer and Health cohort was based on written informed consent.

### Study population

The study was based on the Diet, Cancer and Health cohort [13]. In total, 57,053 of 160,725 residents of Copenhagen or Aarhus aged 50–64 years without a history of cancer were enrolled into the cohort between 1993 and 1997. Participants had to be born in Denmark. At enrolment, each participant completed self-administered, interviewer-checked, questionnaires covering food intake, lifestyle habits including detailed information on present and previous smoking and physical activity, health status including self-reported information on diabetes and social factors. Also, trained staff members measured brachial artery blood pressure at enrolment using automated TAKEDA UA 751 or UA 743 Oscillometers once. The measurement was conducted in the supine position after a minimum of 5 minutes rest and at least 30 minutes after tobacco smoking and intake of food, tea or coffee. If systolic BP was 160 mm Hg or more, or diastolic BP was 95 mm Hg or more, the measurement was repeated after an interval of at least 3 min and the lowest measurement of the two was used. Also, non-fasting total cholesterol (mmol/L) of each individual was determined in whole blood on the day of enrolment using a Lipotrend® C device with Lipotrend test strips (Boehringer Mannheim). The sample was applied onto the test strip by means of a plastic capillary. The study was conducted in accordance with the Helsinki Declaration.

### Identification of outcome

The endpoint was incident MI (International Classification of Disease (ICD) 10: I21.0-I21.9), and potential cases were identified by linkage to the Danish National Hospital Registry [14] and the Danish Causes of Death Registry [15]. Furthermore, we included participants with a sudden cardiac death diagnosis (ICD 10: I46.0-I46.9) if cardiac arrest after validation by medical records was believed to be caused by an MI. From baseline through 2003 potential cases were validated by direct review of medical records in accordance with the guidelines of the American Heart Association and the European Society of Cardiology for use in epidemiology [16]. From January 2004 until end of follow-up (27 June 2006) and for participants whose medical records were not available for review in the period from 1993–2003, we accepted all participants with a diagnosis of MI (ICD 10: I21.0-I21.9) from a ward as cases without further validation, because these diagnoses have been found to have a positive predictive value above 90% in the Danish National Patient Registry [16]. Fatal MI was defined as death within 30 days.

### Exposure assessment

A complete residential address history between 1988 and event or censoring date was collected for 93 % of the cohort members. Exposure to road traffic noise was calculated for the years 1990, 1995, 2000 and 2005 using SoundPLAN (version 6.5, http://www.soundplan.dk/) for all present and historical residential addresses at which these cohort members had lived between 1988 and event/censoring. This noise calculation program implements the joint Nordic prediction method for road traffic noise, which has been the standard method for noise calculation in Scandinavia during many years [17].

The input variables for the noise model were: point for noise estimation (geographical coordinates and height of the floor); road links with information on annual average daily traffic, vehicle distribution, travel speed and road type (motorway, express road, road wider than 6m, road less than 6m and more than 3m, and other road); and building polygons for all buildings, including information on building height. We obtained traffic counts for all Danish roads with more than 1000 vehicles per day from the Danish municipalities as well as from a central database covering all the major state and county roads. We assumed that the terrain was flat, which is a reasonable assumption in Denmark, and that urban areas, roads and areas with water were hard surfaces whereas all other areas were acoustically porous. No information was available on noise barriers. Road traffic noise was calculated as the equivalent continuous A-weighted sound pressure level (L_Aeq_) at the most exposed facade of the dwelling at each address for the day (L_d_; 07:00–19:00 h), evening (L_e_; 19:00–22:00 h) and night (L_n_; 22:00–07:00 h) and expressed as L_den_ by applying a 5-dB penalty for the evening and a 10-dB penalty for the night.[18] Similar to a previous study we used a cut-off value of 42 dB [9], as we considered this a lower limit of ambient noise.

Exposure to railway noise was calculated with the joint Nordic prediction method for railway noise based on general information about rail traffic in 1993–2000. Screening by designated noise screens and buildings was not considered. The noise impact from airports and airfields was determined from information about noise zones (5 dB categories) obtained from local authorities. The curves for railway and aircraft noise were transformed into digital maps and linked to each address by geocodes.

The concentration of NO_x_ in the air was calculated with the Danish AirGIS modelling system for each year (1988–2006) at each address at which the cohort members had lived. AirGIS allows calculation of air pollution at a location as the sum of: local air pollution from traffic in the streets, with the Operational Street Pollution Model; the urban background contribution, calculated with a area source dispersion model; and a regional background contribution [19]. Input data for the AirGIS system included traffic data for individual road links for the period 1988–2005, emission factors for the Danish car fleet, street and building geometry, building height and meteorological data [20]. The AirGIS system has been successfully validated and applied in several studies [21]–[23]. We focused on the concentration of NO_x_ as indicator for air pollution from traffic because measured NO_x_ correlates strongly with other traffic-related pollutants in Danish streets: r = 0.93 for total particle number concentration (10–700 nm) and r = 0.70 for PM_10_ [24], [25].

### Statistical methods

The analyses were based on a Cox proportional hazards model with age as the underlying time [26]. This ensured comparison of individuals of the same age. We used left truncation at age of enrolment, so that people were considered at risk from enrolment into the cohort, and right censoring at the age of MI (event), death, emigration or end of follow-up (27 June 2006), whichever came first. Exposure to road traffic noise and NOx were modelled as time-weighted averages the preceding 5-years (taking all present and historical addresses in that period into account) at a given age or as the yearly exposure at the residence at a given age. These exposures (1- and 5-years) were entered as time-dependent variables into the statistical risk model, thus for each incident MI recalculating exposure for all cohort members at exactly the same age as the case and at risk at the time of the MI.

The incidence rate ratios (IRRs) for MI in association with road traffic noise were calculated for 1) yearly road traffic noise at diagnosis, and 2) time-weighted mean road traffic noise 5-year preceding diagnosis. Estimates were calculated crude and adjusted for *a priori* defined potential confounders: sex, baseline information on smoking status (never, former, current), smoking intensity (1–14, 15–24 and ≥25 g tobacco/day), smoking duration (years), intake of fruit (g/day), intake of vegetables (g/day), education (< 8, 8–10, >10 years), alcohol intake (g/day), body mass index (BMI, <18, 18–25, 26–30≥30 kg/m^2^) and physically active (not active, ≤3.5,>3.5 h/week), information on calendar year, and information on covariates based on address specific information: railway and airport noise (mainly based on data from the late 1990'ies (railway noise > 60 dB (yes/no) and airport noise > 60 dB (yes/no)); and on air pollution (NO_x_, µg/m^3^, data on exposure at the residence in the period from 1988–2006, calculated as 1) yearly mean at the residential address of diagnosis, and 2) 5-year time-weighted mean preceding diagnosis, such that in each analysis the exposure calculation for NO_x_ are similar to the exposure calculation for road traffic noise). In a third model we further adjusted for baseline measured diastolic and systolic blood pressure (mm Hg), measured total cholesterol (mmol/L) and self-reported diabetes. Traffic noise, air pollution, smoking duration, intake of fruits and vegetables, systolic and diastolic blood pressure and total cholesterol were entered as linear variables. Intake of alcohol was included as a spline with a cut-point at 3 g/day. The potential modifiers of an effect between road traffic noise and MI by *a priori* selected baseline confounders and railway noise were evaluated by introducing interaction terms into the model and tested by the Wald test.

**Figure 1:**
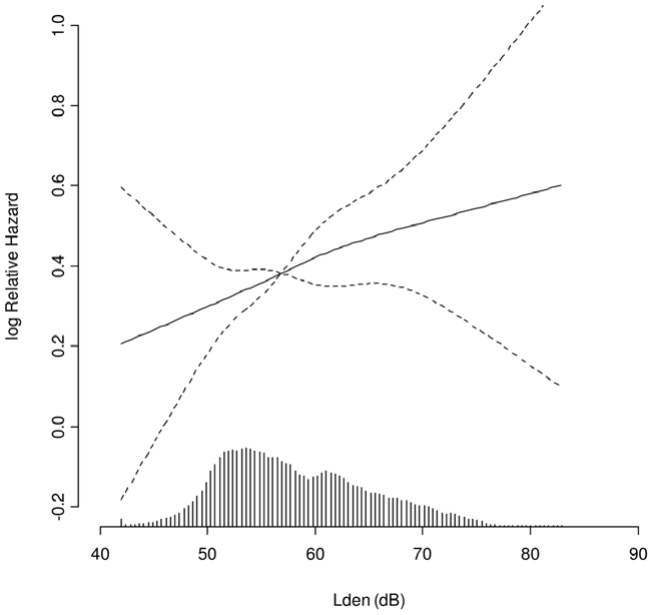
Association between road traffic noise and myocardial infarction. Association between exposure to road traffic noise (L_den_) at the residence at the time of diagnosis and incident MI, adjusted for sex, smoking status, smoking duration, smoking intensity, intake of fruit, vegetables and alcohol, BMI, physical activity, calendar year, education, railway and airport noise, and air pollution. Solid line: incidence rate ratio, dashed lines: 95% confidence interval. The median (56.4 dB) is the reference. The columns at the x-axis show the distribution of exposure to road traffic noise.

The procedure PHREG in SAS version 9.1 (SAS Institute, Cary, North Carolina, USA) was used for the statistical analyses. The exposure-response curve (Figure 1) was estimated and visualized using restricted cubic splines (*library Survival* and *library Design*) in R statistical software 2.9.0.

## Results

Among the 57 053 cohort members, 566 were excluded due to cancer before enrolment and 900 because they had a known endpoint diagnosis (coronary artery disease) at enrolment. Among the remaining 55 587 participants, a complete residential address history between January 1988 and event or censoring date was collected for 52 810 participants. Of these we excluded 2196 participants due to missing data on one or more co-variates leaving a study population of 50 614 participants, including 1600 cases of incident MI. Average follow-up was 9.8 years.

The distribution of road traffic noise exposure (L_den_) is shown in Figure 1. L_den_ at the enrolment address and the time-weighted 5-year mean L_den_ preceding enrolment (taking historical addresses into account) was strongly positively correlated (R_Spearman_ = 0.96, *p*<0.0001). Also, there was a significant positive correlation between L_den_ and NO_x_ in the study period (R_Spearman_ = 0.62, *p*<0.0001). During the period from 1988 to end of follow-up in 2006 there was a small but steady increase over time in L_den_ for the addresses used in this study (0.5 dB increase per 5 years). For NO_x_, the concentration decreased approximately 35% from 1988 to 2000 after which it stabilised.

Cases had higher BMI and lower education, smoked more, drank more alcohol, ate less fruit and vegetables, were less physically active, had higher blood pressure and total cholesterol, had higher prevalence of diabetes and were exposed to more traffic noise and air pollution as compared to the whole cohort (Table 1). Participants living at residences with more than 60 dB from road traffic noise at baseline had higher BMI and lower education, smoked more, ate less fruit and vegetables, were less physically active and had higher prevalence of diabetes compared to participants living with less than 60 dB (Table 1).

**Table 1:** Baseline characteristics by incident myocardial infarction status and by baseline exposure to road traffic noise below and above 60 dB (L_den_).

	Cohort	Cases	L_den_ ≤60	L_den_ >60
Characteristic at enrolment	(N = 50 614)	(N = 1600)	(N = 33 325)	(N = 17 289)
Men, %	48	74	48	46
Age, y	56.1 (50.7–64.2)	58.8 (51.1–64.7)	56.0 (50.7–64.1)	56.3 (50.8–64.2)
BMI, kg/m^2^	25.5 (20.4–33.2)	26.8 (21.1–34.6)	25.4 (20.5–33.0)	25.7 (20.3–33.8)
Years of education, %				
≤7	33	45	31	36
8–10	46	39	47	46
>10	21	16	22	18
Smoking status, %				
Never	36	19	38	32
Former	28	25	29	27
Current 1–14 g/day	13	15	13	14
Current 15–24 g/day	16	27	15	19
Current ≥25 g/day	7	14	6	8
Smoking duration, y a	32 (6–46)	37 (10–49)	32 (6–46)	33 (7–46)
Drink alcohol, %	98	96	98	97
Alcohol intake, g/day	13.4 (1.14–64.7)	15.0 (1.00–69.4)	13.4 (1.20–62.2)	13.3 (1.00–69.6)
Fruit intake, g/day	169 (27.0–521)	147 (19.0–479)	172 (29.0–515)	163 (24.3–533)
Vegetable intake, g/day	162 (49.2–366)	136 (40.8–337)	165 (51.9–366)	155 (44.5–368)
Physically active				
No, %	24	32	23	25
Yes, ≤ 3.5 h/week, %	41	37	42	38
Yes, > 3.5 h/week, %	36	31	35	37
Self-reported diabetes, %	2.0	5.4	1.8	2.4
Systolic blood pressure, mm Hg	138 (109–176)	148 (116–188)	138 (110–175)	139 (109–177)
Diastolic blood pressure mm Hg	83 (67–101)	87 (70–108)	83 (67–101)	83 (67–101)
Total cholesterol, mmol/L	6.0 (4.4–8.2)	6.3 (4.5–8.6)	6.0 (4.4–8.1)	6.1 (4.4–8.2)
Road traffic noise, dB	56.4 (48.5–70.1)	57.7 (48.8–70.6)	53.6 (47.6–59.2)	64.5 (60.4–73.0)
Air pollution, NO_x_, µg/m^3^	20.8 (14.4–88.0)	21.8 (14.4–103)	18.5 (14.2–28.2)	34.2 (16.9–137)

Values are medians (5–95 percentiles) unless otherwise stated.

a Among present and former smokers.

A 10-dB higher level of road traffic noise was associated with a statistically significant 12 % higher risk for MI after adjustment for various risk factors including exposure to NO_x_ for both exposure windows (diagnosis and 5-years preceding diagnosis; Table 2), showing a linear dose-response relationship throughout the exposure range of this study (42–84 dB, Figure 1). Adjustment by NO_x_ only resulted in small changes in estimates: 1.10 (95% CI: 1.03–1.19) before and 1.12 (95% CI: 1.02–1.22) after adjustment for NO_x_ per 10 dB higher L_den_ at diagnosis. There were no significant associations between exposure to NO_x_ at diagnosis or 5-years preceding diagnosis and MI, though the results indicated positive trends. The association between road traffic noise and MI was still present after further adjustment for measured blood pressure and cholesterol and self-reported diabetes (Table 2). We found no significant effect modification, though there were indications of a stronger association with road traffic noise among participants above 65 years and among never smokers (Table 3).

**Table 2:** Incidence rate ratios (IRRs) of MI per 10 dB higher level of exposure to road traffic noise based on 50 614 cohort participants.

Exposure to road traffic noise, L_den_ (per 10 dB)		Adjusted for age	Fully adjusted a	Fully adjusted a + diabetes and measured blood pressure and cholesterol
	N cases	IRR (95% CI)	IRR (95% CI)	IRR (95% CI)
L_den_ at diagnosis	1600	1.14 (1.06–1.23)	1.12 (1.02–1.22)	1.10 (1.00–1.20)
L_den_ 5-year preceding diagnosis	1600	1.14 (1.06–1.23)	1.12 (1.02–1.23)	1.10 (1.00–1.21)

IRR, incidence rate ratio; CI, confidence interval; dB, decibel.

a Adjusted for age, sex, smoking status, smoking duration, smoking intensity, intake of fruit, intake of vegetables, BMI, alcohol intake and physical activity, calendar year, education, railway and airport noise and residential exposure to NO_x._

**Table 3:** Modification of associations between yearly road traffic at the residential address at time of diagnosis and risk for incident MI by baseline characteristics and age at diagnosis.

Co-variates	N cases	IRR (95% CI)^a^	*p interaction*
Sex			0.37
Men	1188	1.14 (1.03–1.26)	
Women	412	1.06 (0.91–1.23)	
Age at MI (years)			0.10
<65	871	1.06 (0.95–1.18)	
≥65	729	1.19 (1.06–1.34)	
Smoking status			0.11
Never	313	1.24 (1.05–1.47)	
Former	398	0.99 (0.85–1.15)	
Current	888	1.14 (1.02–1.27)	
Years of education			0.41
≤7	725	1.07 (0.95–1.20)	
8–10	626	1.14 (1.01–1.30)	
>10	249	1.22 (1.00–1.47)	
Exposed to railway noise ≥60 dB			0.83
Yes	258	1.10 (0.90–1.33)	
No	1342	1.12 (1.02–1.23)	

IRR, incidence rate ratio; CI, confidence interval; dB, decibel.

a Adjusted for age, sex, lifestyle confounders (smoking status, smoking duration, smoking intensity, intake of fruit, vegetables and alcohol, BMI and physical activity), calendar year, education, railway and airport noise, and air pollution (NO_x_).

Among the 331 cases with a fatal MI, the IRR per 10 dB yearly road traffic noise at the residential address at diagnosis was 1.25 (95% CI: 1.07–1.46) in the crude analysis and 1.17 (95% CI: 0.96–1.43) in the fully adjusted analysis.

## Discussion

In this study residential exposure to road traffic noise was associated with a 12% higher risk for incident MI per 10 dB higher exposure to noise showing a clear dose-response relationship.

A case-control study has previously investigated effect of long-term road traffic noise during a period of more than 20 years on risk of having a MI after adjustment for air pollution, assessed as modelled NO_2_ [9]. They found that exposure to road traffic noise of 50 dB or more resulted in a 12% (95% CI: −5% to 33%) higher risk for MI, which is similar to the present study. Unlike the present study, they found only a weak suggestion of a dose-response relationship. However, after excluding persons with hearing loss and exposure to noise from other sources they found that noise exposure of 50 or more dB was associated with a 38% (95% CI: 11% – 71%) higher risk for MI, with a significant linear trend over noise categories. Especially exclusion of persons exposed to railway noise seemed to result in higher estimates [9], which is unlike the present study with similar risks among persons exposed to over and under 60 dB of railway noise.

In a cohort study by Beelen et al. the joint effect of road traffic noise and air pollution, assessed as black smoke and traffic intensity, on mortality from ischemic heart disease was investigated [12]. They found a tendency of a weak association only in the highest exposure category (>65 dB), which disappeared after adjustment for air pollution. These findings are in contrast to the previous [9] and the present study which both found that road traffic noise increased the risk for fatal MI, with slightly higher risk estimates for fatal MI as compared with all MI. A possible explanation is that the previous [9] and the present study focused on ‘acute MI’ (ICD10: I20) whereas the study by Beelen et al. had a more broad case definition, covering e.g. heart aneurysms (ICD10: I20-I25). Therefore, it seems possible that road traffic noise mainly have an effect on incident MI. Also, Beelen et al. used baseline addresses to model exposure whereas both Selander et al. and the present study used historical address history. Finally, Beelen et al. adjusted not only for air pollution (black smoke) but also for traffic intensity which is a major determinant of the noise level. This could have resulted in over-adjustment of the noise effect. In contrast, the present study and Selander et al. adjusted for NO_x_ and NO_2_, respectively, both modelled using dispersion models that calculate air pollution as the sum of local, urban background and regional background contributions [19], and both with a moderate R^2^ between road traffic noise and indicator of air pollution on around 0.36. In the present study adjustment for NO_x_ only caused small changes in the estimates suggesting an independent effect of road traffic noise on MI.

Hypertension, hypercholesterolemia and diabetes are established risk factors for MI but also outcomes suspected to be associated with exposure to traffic noise [27], and therefore possibly on the biological pathway between exposure to traffic noise and a MI. In the present study, adjustment for measured blood pressure, measured total cholesterol and self-reported diabetes only resulted in minor attenuations in estimates, indicating that other pathways are involved in the effect of traffic noise on risk for MI.

Some studies have similar to the present study indicated that the effect of traffic noise (road and aircraft) on cardiovascular disease are strongest among men [28]–[30], whereas others have indicated no sex differences [9], [10], [12]. Also with regard to effect modification by age the results are inconclusive, as similar to the present study strongest effects of traffic noise have been found among the oldest participants in relation to stroke [10] and MI [29], whereas other studies indicated no effect modification by age in relation to MI [9] and cardiovascular mortality [12]. Sleep disturbances can contribute to cardiovascular risk [31], [32], leading to the hypothesis that exposure to noise during night might be more harmful than daytime exposure. The sleep structure generally becomes more fragmented with age, and elderly people are thus more susceptible to sleep disturbances [33], [34].

Stress and sleep disturbances can cause changes lifestyle habits, including increased tobacco smoking, and thus potentially a stronger association between traffic noise and MI among smokers. However, we found indications of a high effect of road traffic noise on MI among never smokers, with no clear pattern with regard to smoking status. Other similar studies are inconclusive as one study indicated effect of road traffic noise on MI only among former smokers [9], whereas no effect modification by smoking was found for road traffic noise on cardiovascular mortality [12].

Both short- and long-term exposure to traffic noise may affect the risk for MI. Risk factors for development of arthrosclerosis include hypertension, inflammation and glucose intolerance, which have all been associated with traffic noise, stress and/or sleep disturbances [27], [35]–[37]. On the other hand, inflammation and high blood pressure may also have more acute effects, including effect on plaque rupture. We investigated effects of both relatively recent exposure, namely yearly noise, and longer term exposure, namely 5-year noise. The results were virtually identical, probably due to that only 28% moved during follow-up causing a strong correlation between recent and more distant exposure. It is therefore impossible for us to separate the effect of recent and distant noise exposure in relation to MI.

The strengths of our study include the prospective design, the large number of cases, the strict validation of the diagnosis of MI using nationwide registers and review of medical records, and access to residential address histories. Furthermore, we considered only the first hospitalization for MI, reducing any influence of preventive medication on the risk estimates. Another strength is adjustment for air pollution, which is known to correlate with road traffic noise and, thus, could confound the association between noise and MI. We used NO_x_ levels as an indicator of air pollution, because NO_x_ is a good marker of traffic-related air pollution and correlates closely with particulate matter, both ultrafine particles and PM_10_ [25]. Long-term exposures to air pollution has been associated with risk of ischemic heart disease, strongest for fatal events [11]. We found that exposure to road traffic noise was significantly associated with risk for MI both before and after adjustment by air pollution, suggesting an independent effect of road traffic noise.

Some limitations also need to be considered. Our study is not representative of the Danish population as the study population lived mainly in urban areas. Also, death from other causes that MI results in censoring, which excludes participants dying from other cardiovascular disease associated with traffic noise such as stroke, and potentially with high risk of MI if they had survived. This could have underestimated the true association. The estimation of noise was based on modelled values. Although the Nordic prediction method has been used for many years, estimation of noise is inevitably associated with some degree of uncertainty. One reason could be inaccurate input data, which would result in exposure misclassification. As the noise model does not distinguish between cases and the cohort, such misclassification is believed to be non-differential, and, in most situations, this would influence the relative risk estimate towards the neutral value. Also, we had information only on residential addresses. Such an imprecision is, however, believed to have been similar for cases and cohort and might therefore have attenuated the estimates. We also had no information on bedroom location, noise from neighbours and ventilation or hearing impairment, all of which might influence exposure to noise. The study by Selander et al. investigating effects of road traffic noise on incident MI found that the association with road traffic noise was stronger when several of these factors were considered [9], suggesting that the effect of noise might be underestimated in the present study. Finally, there might be residual confounding from risk factors not accounted for in the analyses, such as familiar history of MI. However, adjustment of our analyses resulted in only minor changes in the estimates although we adjusted for many important risk factors for MI, and, therefore, residual confounding seems not to be a major issue in the present study.

In conclusion, the present study shows a positive association between residential exposure to road traffic noise and risk for MI in a general Danish population, with a clear dose-response relationship.
